# Conversion of Methionine to Cysteine in *Lactobacillus paracasei* Depends on the Highly Mobile *cysK-ctl-cysE* Gene Cluster

**DOI:** 10.3389/fmicb.2018.02415

**Published:** 2018-10-17

**Authors:** Daniel Wüthrich, Stefan Irmler, Hélène Berthoud, Barbara Guggenbühl, Elisabeth Eugster, Rémy Bruggmann

**Affiliations:** ^1^Interfaculty Bioinformatics Unit and Swiss Institute of Bioinformatics, University of Bern, Bern, Switzerland; ^2^Agroscope, Bern, Switzerland; ^3^School of Agricultural, Forest and Food Sciences HAFL, Bern University of Applied Sciences, Zollikofen, Switzerland

**Keywords:** horizontal gene transfer, gene cluster, methionine, cysteine, *Lactobacillus paracasei*, core genome, pan genome, milk

## Abstract

Milk and dairy products are rich in nutrients and are therefore habitats for various microbiomes. However, the composition of nutrients can be quite diverse, in particular among the sulfur containing amino acids. In milk, methionine is present in a 25-fold higher abundance than cysteine. Interestingly, a fraction of strains of the species *L. paracasei* – a flavor-enhancing adjunct culture species – can grow in medium with methionine as the sole sulfur source. In this study, we focus on genomic and evolutionary aspects of sulfur dependence in *L. paracasei* strains. From 24 selected *L. paracasei* strains, 16 strains can grow in medium with methionine as sole sulfur source. We sequenced these strains to perform gene-trait matching. We found that one gene cluster – consisting of a cysteine synthase, a cystathionine lyase, and a serine acetyltransferase – is present in all strains that grow in medium with methionine as sole sulfur source. In contrast, strains that depend on other sulfur sources do not have this gene cluster. We expanded the study and searched for this gene cluster in other species and detected it in the genomes of many bacteria species used in the food production. The comparison to these species showed that two different versions of the gene cluster exist in *L. paracasei* which were likely gained in two distinct events of horizontal gene transfer. Additionally, the comparison of 62 *L. paracasei* genomes and the two versions of the gene cluster revealed that this gene cluster is mobile within the species.

## Introduction

*Lactobacillus paracasei* belongs to the LAB, which are gram-positive bacteria with lactic acid as the main product of the carbohydrate metabolism. It must be noted that the taxonomic status of *L. paracasei* has been debated during recent years (Collins et al., 1989; Wayne, 1994; Judicial Commission of the International Committee on Systematics of Bacteria, 2008). Consequently, in many cases, strains described as *Lactobacillus casei* are more closely related to *L. paracasei* ATCC 334 (previously known as *L. casei* ATCC 334) than to the species type strain ATCC 393. In this report, we use the species name *L. paracasei*, because the strains used in this study are more closely related to *L. paracasei* ATCC 334 (mean genome similarity 98.6%) than *L. casei* ATCC 393 (mean genome similarity 78.3%).

*Lactobacillus paracasei* bacteria are found in various habitats, such as plants, human body, and fermented food (Hammes and Hertel, 2006). It was suggested that the species *L. paracasei* has a large gene pool that allows the bacteria to adapt to the different habitats (Broadbent et al., 2012; Smokvina et al., 2013). The large gene pool is an asset for industrial applications and in particular for the dairy industry. Contrary to the natural habitats, the emergence of the milk and cheese as habitats is a rather recent event (Salque et al., 2013). These habitats are rich in nutrients like carbohydrates, proteins and lipids compared to natural habitats. However, the diversity of these nutrients is rather limited, as the main constituents of carbohydrates and proteins are lactose and caseins, respectively. Furthermore, in cheese the diversity of bacteria is high (Quigley et al., 2012) and therefore, the competition pressure is high which might be an advantage for fast growing bacteria with small genomes. It was proposed in earlier studies that the adaptation to the milk related habitats caused gene loss in *L. paracasei* (Cai et al., 2009).

The sulfur containing amino acids methionine and cysteine are a good example for the specificity of milk as a habitat. Bogicevic et al. (2013) described that the methionine concentration excesses the cysteine during the cheese ripening upto a factor of 25. Therefore the ability to convert methionine to cysteine is an important growth advantage. Several of the genes involved in the methionine and cysteine metabolism are already described in *L. paracasei*. It was shown that genes coding for cystathionine lyase are present in the genomes of several *L. paracasei* strains (Irmler et al., 2009). It was also shown that an O-acetylhomoserine sulfhydrylase (cysteine synthase) is coded in the genomes of many strains (Bogicevic et al., 2012a). Recent findings showed that a gene cluster of a cysteine synthase, a cystathionine lyase and a serine acetyltransferase (*cysK-ctl-cysE* gene cluster) is involved in the sulfur metabolism in *L. paracasei* (Liu et al., 2008; Bogicevic et al., 2012b). However, the genomic and evolutionary basis to convert methionine to cysteine has not been studied in detail in *L. paracasei*.

The aim of this study was to find genomic elements that allow *L. paracasei* strains to convert methionine to cysteine and to study their evolutionary origin. Therefore, we investigated strain specific genes using whole genome sequencing and tested if the strains can grow with methionine as the sole sulfur source. With the resulting data, we performed gene-trait matching to find the phenotype causing genes. We found that the previously described gene cluster *cysK-ctl-cysE* (Bogicevic et al., 2012b) is the genomic element that allows a subpopulation of the *L. paracasei* strains to synthetize cysteine from methionine. Furthermore, we found evidence that the gene cluster is very mobile, and was possibly gained twice by the species by horizontal gene transfer and is mobile within the species.

## Results

### Sequencing and Genome Assembly

We sequenced the genomes of 40 *L. paracasei* strains from the Agroscope culture collection originating from dairy products to an average depth of coverage of about 1200× (**Supplementary Table S1**). In addition, we used the long-read technology of Pacific Biosciences to sequence six bacterial strains (FAM18149, FAM18121, FAM10859, FAM18099, FAM18101, and FAM3228) selected from the 40 strains. These six nearly complete genomes were used to order the contigs of the other genomes which allowed us to better perform comparative genomics tasks. It also allowed us to identify if a strain has plasmids. We applied a hybrid assembly with Illumina short reads and PacBio long leads, as the long reads generated with the PacBio C2 chemistry do not allow the use of the non-hybrid HGAP method (Chin et al., 2013). Nevertheless, we obtained almost complete genome assemblies, which consist of only a few scaffolds (3–21 scaffolds, **Supplementary Table S1**). The remaining 34 strains were assembled using Illumina short reads only. The median n50 of the assemblies is 63,528 bp which is comparable with the results of similar studies performed with *L. paracasei* (Broadbent et al., 2012; Smokvina et al., 2013). The scaffolds of the genome assemblies of these 34 strains were ordered according to the six nearly complete genome assemblies as well as publicly available complete genome assemblies. We performed *ab initio* annotation of all 40 genomes to identify the CDSs using Prokka (Seemann, 2014). The number of CDSs per genome ranges from 2,501 (FAM18132) to 3,078 (FAM3257) with a median of 2,930 CDSs (**Supplementary Table S1**).

### Gene-Trait Matching

**FIGURE 1:**
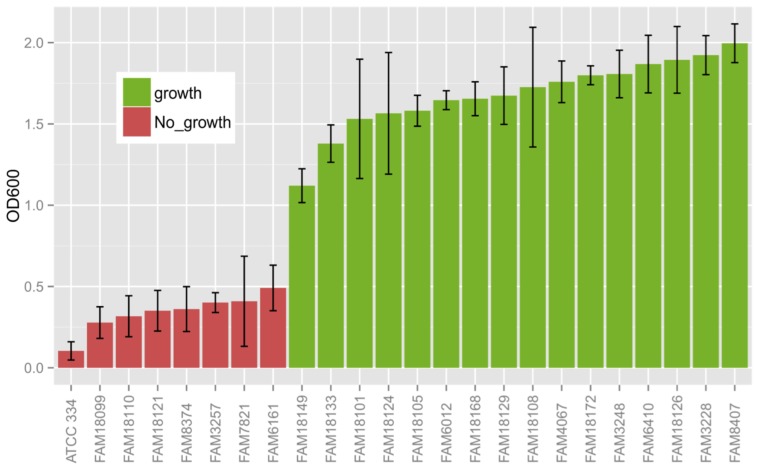
Growth of various *L. paracasei* strains in a chemically defined medium with methionine as the sole sulfur source. The bars and black lines represent the mean and standard deviation of the reached OD600 after 25 h of growth, respectively, of three independent biologically repeated measurements. Strains that reached an OD600 of one or more were consider as being able to grow in a medium with methionine as sole sulfur source.

We tested 23 sequenced strains and the reference strain ATCC 334 of *L. paracasei* (Makarova et al., 2006) if they can grow in medium with methionine as sole sulfur source. We incubated the strains for 25 h in CDM with methionine as sole sulfur source and measured the OD600 of the 24 strains. The experiment was performed in triplicates. Strains that reached a mean OD600 of one or higher were considered as growth positive. In total, 16 out of 24 strains could grow under this condition (**Figure 1**). The strains were also grown in medium containing cysteine and methionine and all grew to an OD600 > 1 (data not shown). To compare the phenotype with the genotype, we first determined OGCs shared between the strains using OrthoMCL (Li et al., 2003). In total, we found 5,406 OGCs within the 24 strains. We tested all 5,406 OGCs using Fisher’s exact test (Fisher, 1922) for overrepresentation in strains that can or cannot grow with methionine as sole sulfur source. This resulted in three OGCs that are significantly (p-adj. < 0.05) overrepresented in strains that can grow with methionine as sole sulfur source. These three genes build the *cysK-ctl-cysE* gene cluster and are neighboring in all 16 strains that can grow with methionine as sole sulfur source. The *cysK-ctl-cysE* gene cluster contains a cysteine synthase, a cystathionine lyase and a serine acetyltransferase (Bogicevic et al., 2012b). Worth mentioning is the fact that we found an ortholog of cysE in the strain FAM3257 that cannot grow without cysteine, whereas cysK and ctl are exclusively present in strains that can grow without cysteine.

### Phylogeny of the *cysK-ctl-cysE* Gene Cluster

**FIGURE 2:**
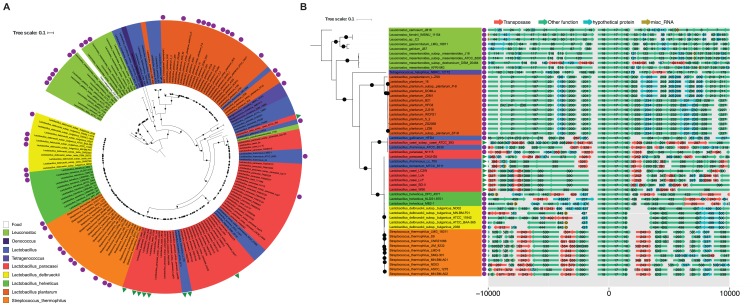
The phylogeny and the genomic context of the *cysK-ctl-cysE* gene cluster. **(A)** The phylogenetic tree shows the relationship between the gene clusters of the different strains. The black dots indicate a bootstraps support of 80% or more. The *cysK-ctl-cysE* gene cluster was found in various species. Gene clusters identified on a completely assembled chromosome were indicated with a purple circle and gene clusters identified on a complete plasmid are indicated with a green triangle. Panel **(B)** depicts the genomic context (± 10,000 bp) of the gene clusters found on a completely assembly plasmid or chromosome. The function of the genes is represented by the colors of the arrows. The numbers on the arrows indicate the orthologous groups the genes belong to.

To study the evolutionary history of the *cysK-ctl-cysE* gene cluster, we extended our dataset of 24 strains with 16 additional strains from the Agroscope strain collection in order to have a larger dataset. We searched the 40 sequenced *L. paracasei* genomes and the non-redundant nucleotide databases from GenBank for this gene cluster. Of the 40 strains, 11 strains carried the *cysK2-ctl1-cysE2* and 13 strains carried the *cysK3-ctl2-cysE3* gene cluster. In addition, we identified the gene clusters in 166 different nucleotide sequences of the non-redundant nucleotide database from GenBank, which were mainly from species found in fermented food. We built a phylogenetic tree of these sequences and found that the gene cluster of *L. paracasei* is spread over two separate branches (**Figure 2A**). A previous study described that two versions of the gene cluster exist in *L. paracasei* and they were called *cysK2-ctl1-cysE2* and *cysK3-ctl2-cysE3* (Bogicevic et al., 2012b), respectively. These do correspond to the gene cluster we have identified. Interestingly, *cysK2-ctl1-cysE2* clusters with the gene clusters of *S. thermophilus*, *L. helveticus*, *L. fermentum*, and *L. delbrueckii*, whereas *cysK3-ctl2-cysE3* clusters with the ones of *L. rhamnosus*, *L. casei*, *L. gallinarum*, and *L. pseudomesenteroides*. From the 166 sequences containing the gene cluster, 65 are from a completed chromosome or plasmid assembly. Interestingly, only the clusters that are closely related with *cysK2-ctl1-cysE2* and in *L. casei* ATCC 393 (*cysK3-ctl2-cysE3*) are located on a plasmid (**Figure 2A**).

To study the genomic environment of the gene cluster in the different species, we depicted all neighboring genes of all complete chromosomes and plasmids (**Figure 2B**). The figure shows that the neighboring genes are highly conserved, if the clusters are closely related. However, we also found that the gene cluster has many neighboring transposases in most *Lactobacillus* and *Streptococcus* species. In contrast, in the *Leuconostoc* and *Lactobacillus plantarum*, we found that almost no transposases are in close proximity to the gene cluster.

### The Phylogeny of *L. paracasei*

**FIGURE 3:**
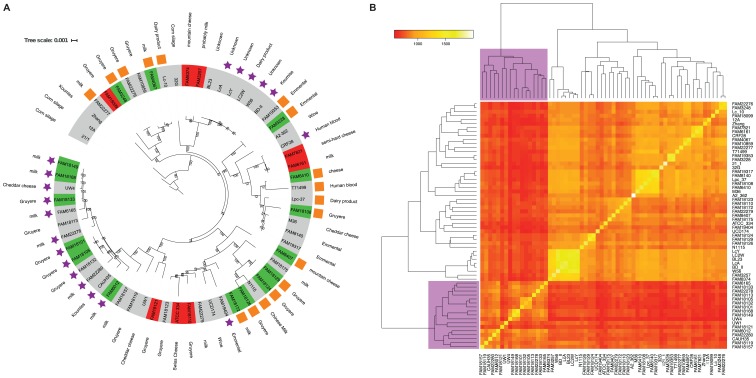
Comparison of phylogeny and gene content. **(A)** The branch length indicates the phylogenetic distance. The numbers at the branching events indicate the bootstrap support. Strains with the *cysK2-ctl1-cysE2* gene cluster are labeled with a purple star, the strains with the *cysK3-ctl2-cysE3* gene cluster are labeled with an orange square. The colors of the strain names show if the strain is able (green) or unable (red) to grow with methionine a sole sulfur source. Strain that were not measured are labeled in gray. **(B)** The colors of the different fields in the heat map represent the numbers of shared GCs of two strains of the accessory genome. Based on these numbers, the Euclidean distance was calculated to build the dendrogram. The dendrogram separates the strains into group 1 and group 2. The purple square indicates the strains located on the phylogenetic branch where the strains carry *cysK2-ctl1-cysE2* gene clusters.

As we found two different versions of the *cysK-ctl-cysE* gene cluster, we studied the correlation of the presence of the two versions of the gene cluster with the phylogenetic relation of the strains. We extended our 40 strains with 17 published genomes from strains derived from diverse habitats (Broadbent et al., 2012) and the genomes of five previously published *L. paracasei* genomes, which are completely assembled and have a *cysK-ctl-cysE* gene cluster. We built GCs for all translated CDSs of all 62 strains using Roary (Page et al., 2015). Roary is more sensitive regarding paralog separation than OrthoMCL. Interestingly, the genes of the *cysK2-ctl1-cysE2* and *cysK3-ctl2-cysE3* gene clusters are not considered as orthologs in this analysis. In total, the size of the pan genome is 11,613 GCs and the size of the core genome is 1,305 GCs. Based on the core genome, we constructed a phylogenetic tree (**Figure 3A**). We found that most strains that carry the *cysK2-ctl1-cysE2* version of the gene cluster are located on a separated branch or appear in some single strains. The strains LcA, LcY, LC2W, W56, and BD-II do also carry the *cysK2-ctl1-cysE2* gene cluster and build their own branch in the phylogenetic tree. However, the phylogenetic distance between these strains is very close and they might even be clones of each other. Worth mentioning is that the strain BL23 is also very closely related to these strains but does not have the gene cluster.

We clustered the 62 strains based on their gene pool to study the diversity of the gene repertoire among the different strains. We constructed a dendrogram of the strains based on the number of shared GCs (**Figure 3B**) using the Euclidean distance as a measure (Warnes et al., 2014). The analysis resulted in a separation of the strains into a large group with 45 strains and smaller group with 17 strains. The comparison of the dendrogram based on the Euclidean distance of the gene content with the phylogenetic tree showed that the 17 strains from the smaller group lie on one branch in the phylogenetic tree (**Figure 3A**), which contains many strains with the *cysK2-ctl1-cysE2* gene cluster. This shows that the strains adapted their genepools during the separation into the two groups.

### Conservation of the Genomic Context of the *cysK-ctl-cysE* Gene Cluster in *L. paracasei*

We identified *cysK-ctl-cysE* as the main genomic element that allows *L. paracasei* to produce cysteine from methionine. To minimize the possibility that we have missed other important genes, we studied genes that might have been transferred within the species along with the *cysK-ctl-cysE* gene cluster. Therefore, we analyzed the surrounding genes of the gene cluster in more detail. As the gene cluster is flanked by transposases (**Figure 2B**), that may lead to breaks in the genome assembly, we searched for orthologs of the neighboring genes of completely assembled strains: BD-II, CAUH35, LC2W, LcA, LcY, and W56 for the *cysK2-ctl1-cysE2* cluster and N1115 for the *cysK3-ctl2-cysE3* cluster.

**FIGURE 4:**
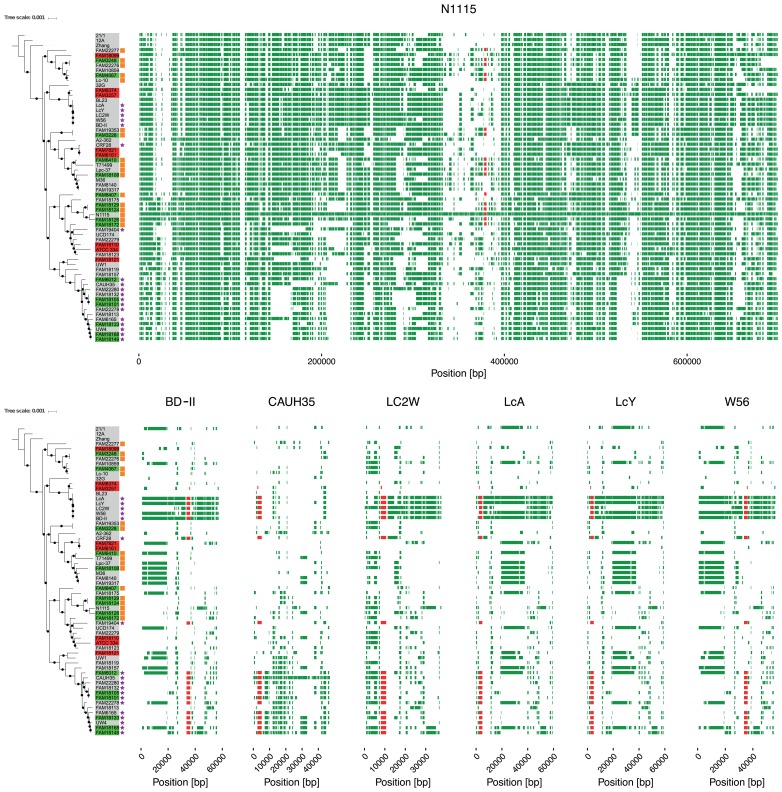
Positions of unconserved regions. Visualization of conservation of the genes around *cysK-ctl-cysE* gene cluster. Orthologs were identified using the paralog aware algorithm Roary (Page et al., 2015). The X-axis represents the genomic position on the chromosome (upper panel) or the position on the plasmid (lower panel) of the different protein coding genes. Each green line represents the genes found in the genomes as ortholog. The position of the *cysK-ctl-cysE* gene cluster is indicated in red. The length of a branch represents the phylogenetic distance. The black dots at the branching events indicate a bootstrap support of at least 80%.

In contrast to the *cysK2-ctl1-cysE2* cluster, the *cysK3-ctl2-cysE3* is located on the chromosome and not a plasmid. For strain N1115, we found that most genes of the surrounding 70 kb are not present in any of the other 61 strains (**Figure 4**). The comparison with the Islandviewer 4 (Langille and Brinkman, 2009) showed that N1115 has predicted genomic islands from 341,661 to 363,478 and a second one from 377,342 to 408,141. Interestingly, we did not find larger parts of the genomic islands within the genomes of any of the other strain, not even in the strains that carry the *cysK3-ctl2-cysE3* gene cluster.

In the phylogenetic analysis, we found that LcA, LcY, BD-II, W56, and LC2W are closely related. The comparison of the plasmids of these five strains shows that not only the gene cluster but also the other genes on the plasmids are conserved within these strains, except for LC2W (**Figure 4**). The plasmid of LC2W is much smaller than the plasmids of the other four strains. This indicates that the plasmid is rather unstable, as the difference in size is enormous between such closely related strains. By expanding the analysis to all strains that carry the *cysK2-ctl1-cysE2*, we found that barely any region of the plasmids is conserved in other strains. Next to the gene cluster also the first 20 kbp are conserved within most strains. Interestingly, a large part of the plasmid is also conserved within strains of the branch that do not carry the gene cluster (UW1, FAM18119, FAM18157, and FAM18113).

## Discussion

To be a successful bacteria species in a medium such as milk, which is rich in the amount of nutrients, but rather limited in its diversity, it is indispensable for the bacterium to metabolize these substrates into essential compounds. This is especially true for sulfur containing amino acids, as it was shown by Bogicevic et al. (2013) that methionine is 25 times more abundant than cysteine during the cheese ripening process. Therefore, the ability to convert methionine to cysteine is a selective growth advantage.

In this study, we found that the *cysK-ctl-cysE* gene cluster is the most important gene cluster that allows *L. paracasei* strains to grow with methionine as sole sulfur source. The gene-trait matching analysis clearly showed that all three genes are required for the conversion of methionine to cysteine and that they are conserved as a unit. We conclude that the gain of the *cysK-ctl-cysE* gene cluster is sufficient for *L. paracasei* strains to acquire the ability to synthesize cysteine from methionine. A previous study proposed this role of the gene cluster for *S. thermophilus* and *L. bulgaricus* (Liu et al., 2009). Additionally, a model was published that explained the role of ctl in the volatile sulfur compounds production (Bogicevic et al., 2013). Finally, we recently published that the *cysK-ctl-cysE* is massively up regulated in *L. paracasei*, if no cysteine is present in the growth medium (Wüthrich et al., 2018).

As we searched for this gene cluster in other species, we found that it is not only present in *L. paracasei*, but also in many species that are also used in the food production. This is more evidence that this gene cluster may be transmitted between species by horizontal gene transfer, as it was already suggested in a previous study (Liu et al., 2009). In *Leuconostoc* species, we found that the gene cluster has almost no flanking transposases and was found in several species in the genus. Also, the genomic context was conserved within the species. Therefore, we propose that the gene cluster is not very mobile within the *Leuconostoc* and ancestor of this genus may also be the origin of this gene cluster. In contrast, all *Lactobacilli*, except for *L. plantarum*, show many transposases in the genomic region of the *cysK-ctl-cysE* gene cluster. We suggest that this may lead to a highly mobile gene cluster.

Within the species *L. paracasei*, we found that two different versions of the cluster exist (**Figure 2A**). We assume that these two versions were introduced in two different events of horizontal gene transfer, as they are closely related to gene clusters in other species and are separated within the phylogenetic tree of the studied *L. paracasei* strains (**Figure 3A**). While the *cysK3-ctl2-cysE3* cluster is found in many branches of the phylogenetic tree, the *cysK2-ctl1-cysE2* gene clusters are located on one branch (**Figure 3A**). We also found the *cysK2-ctl1-cysE2* gene cluster in the strains CRF28, FAM19404 as well as in LcA, LcY, BD-II, W56, and LC2W. Because we found that all identified versions of the *cysK2-ctl1-cysE2* gene cluster are coded on a plasmid and therefore are mobile, we concluded that this gene cluster was horizontally transferred within the species.

The comparison of the gene pool of the strains of the branch with the strains carrying the *cysK2-ctl1-cysE2* gene cluster and the rest of the 62 strains showed that they have a clearly separated gene pool (**Figure 3B**). A similar separation was found in studies about *Oenococcus oeni* (Campbell-Sills et al., 2015) and *Streptococcus pneumoniae* (Hilty et al., 2014) in which the authors concluded that it was induced by an alteration of the habitat. However, with the *cysK2-ctl1-cysE2* gene cluster, we found a good example that there is an exchange of these separated groups by horizontal gene transfer.

Finally, we could show the differences between the two popular ortholog finding algorithms Roary (Page et al., 2015) and OrthoMCL (Li et al., 2003). While the genes of the *cysK2-ctl1-cysE2* and the *cysK3-ctl2-cysE3* were not considered as orthologs using Roary, they were classified as orthologs by OrthoMCL, although, the two genes cluster were not gained in the same evolutionary event. Therefore, we conclude that the strength of Roary is to cluster genes from the same evolutionary events, whereas the strength of OrthoMCL is to identify genes with similar functions (Ding et al., 2018). However, in this study we tested only the default parameters of the algorithms.

## Materials and Methods

### Bacterial Strains, Media, and Growth Conditions

*Lactobacillus paracasei* strains were obtained from the Agroscope culture collection in Liebefeld (Berne, Switzerland) and were maintained in MRS broth (De Man et al., 1960). The requirement for cysteine was assayed by inoculating a chemically defined medium (Christensen and Steele, 2003) devoid of cysteine. Optical density at 600 nm (OD600) was determined with a spectrophotometer (LKB Biochrom 4050 Ultrospec II).

### Library Preparation and Illumina Sequencing

Three different protocols to prepare the sequencing library were used: Nextera DNA Sample Preparation Kit (Art. No. 15028212, Illumina, San Diego, CA, United States); TruSeq DNA Sample Preparation Kit (Art. No. 15025064); and Nugen Encore Rapid DR Multiplex System 1–96 (Art. No. 0328-96). The fragment size was 300–3,000 bp for the Nextera protocol, 400–500 bp for Illumina TruSeq, and 400–1000 bp for Nugen, respectively.

The sequencing was performed on an Illumina HiSeq 2000 (Illumina Inc., San Diego, CA, United States) using TruSeq v3 chemistry.

### Library Preparation and PacBio Sequencing

High molecular weight of DNA from the *L. paracasei* strains was sheared in a Covaris instrument (Covaris, Woburn, MA, United States) to 10 kb fragments, and the DNA size distribution was checked on a fragment analyzer (Advanced Analytical Technologies, Ames, IA, United States). Then, 5 μg of the sheared DNA was used to prepare a SMRTbell library using the PacBio DNA Template Prep Kit 2.0 (Pacific Biosciences, Menlo Park, CA, United States), according to the manufacturer’s recommendations. The library was sequenced using one SMRT cell v2 with C2 chemistry on a PacBio RSII system (Pacific Biosciences, Menlo Park, CA, United States) that was given a movie length of 90 min.

### Genomes

The data (assembled genomes and annotation) of the 40 sequenced strains is available as BioProject under the number PRJNA295910^1^: SAMN04088858, LKFC00000000, FAM10859; SAMN04088859, LKFD00000000, FAM18099; SAMN04088860, LKFE00000000, FAM18101; SAMN04088861, LKFF00000000, FAM18105; SAMN04088862, LKFG00000000, FAM18108; SAMN04088863, LKFH00000000, FAM18110; SAMN04088864, LKFI00000000, FAM18113; SAMN04088865, LKFJ00000000, FAM18119; SAMN04088866, LKFK00000000, FAM18121; SAMN04088867, LKFL00000000, FAM18123; SAMN04088868, LKFM00000000, FAM18124; SAMN04088869, LKFN00000000, FAM18126; SAMN04088870, LKFO00000000, FAM18129; SAMN04088871, LKFP00000000, FAM18132; SAMN04088872, LKFQ00000000, FAM18133; SAMN04088873, LKFR00000000, FAM18149; SAMN04088874, LKFS00000000, FAM18157; SAMN04088875, LKFT00000000, FAM18168; SAMN04088876, LKFU00000000, FAM18172; SAMN04088877, LKFV00000000, FAM18175; SAMN04088878, LKFW00000000, FAM19317; SAMN04088879, LKFX00000000, FAM19353; SAMN04088880, LKFY00000000, FAM19404; SAMN04088881, LKFZ00000000, FAM22276; SAMN04088882, LKGA00000000, FAM22277; SAMN04088883, LKGB00000000, FAM22278; SAMN04088884, LKGC00000000, FAM22279; SAMN04088885, LKGD00000000, FAM22280; SAMN04088886, LKGE00000000, FAM3228; SAMN04088887, LKGF00000000, FAM3248; SAMN04088888, LKGG00000000, FAM3257; SAMN04088889, LKGH00000000, FAM4067; SAMN04088890, LKGI00000000, FAM6012; SAMN04088891, LKGJ00000000, FAM6161; SAMN04088892, LKGK00000000, FAM6165; SAMN04088893, LKGL00000000, FAM6410; SAMN04088894, LKGM00000000, FAM7821; SAMN04088895, LKGN00000000, FAM8140; SAMN04088896, LKGO00000000, FAM8374; and SAMN04088897, LKGP00000000, FAM8407.

Additional genome sequences were retrieved from GenBank^2^. The genomes ATCC_334 (GenBank: NC_008526 and NC_008502), M36 (GenBank: AFYO00000000), UW1 (GenBank: AFYR00000000), UW4 (GenBank: AFYS00000000), Zhang (GenBank: NC_014334 and NC_011352), BD-II (GenBank: CP002618 and CP002619), LC2W (GenBank: CP002616 and CP002617), Lc-10 (GenBank: AFYT00000000), Lpc-37 (GenBank: AFYU00000000), BL23 (GenBank: NC_010999), 12A (GenBank: AFYJ00000000), 21/1 (GenBank: AFYK00000000), 32G (GenBank: AFYL00000000), A2-362 (GenBank: AFYM00000000), UCD174 (GenBank: AFYQ00000000), T71499 (GenBank: AFYP00000000), CRF28 (GenBank: AFYN00000000), W56 (GenBank: NC_018641.1 and NC_020057.1), CAUH35 (GenBank: NZ_CP012187.1, NZ_CP012188.1, NZ_CP012189.1, NZ_CP012190.1, and NZ_CP012191.1), LcA (GenBank: NZ_CM001861.1 and NZ_CM001862.1), LcY (GenBank: NZ_CM001848.2 and NZ_CM002348.1), and N1115 (GenBank: NZ_CP007122.1, NZ_CP007123.1, NZ_CP007124.1, NZ_CP007125.1, and NZ_CP007126.1) were the additional *L. paracasei* strains. The genome of the type strain ATCC393 (GenBank: AP012544.1) of *L. casei* was used.

### Assembly and Annotation

The reads of the TruSeq libraries were assembled using the SPAdes assembly pipeline (version 3.0, options: –careful –mismatch-correction -k 21,33,55,77,89,95,99) (Bankevich et al., 2012). In case PacBio data were available, a hybrid assembly was performed using the SPAdes assembly pipeline (version 3.0, options: –careful –mismatch-correction -k 21,33,55,77,89,95,99 –pacbio) (Bankevich et al., 2012). The accuracy of the assembled contigs was improved using SEQuel (version 1.0.2, default parameters) (Ronen et al., 2012) and was followed by scaffolding with SSPACE (version 2.0, default parameters) (Boetzer et al., 2011) using all reads from the three libraries as input. GapFiller (v1-11, default parameters) (Boetzer and Pirovano, 2012) was applied to all scaffolds. Scaffolds smaller than 500 bp and/or a read-depth below 10% of genome average were excluded. Bowtie2 (version 2.1.0, default parameters) (Langmead and Salzberg, 2012) was used for remapping, and the read-depth was determined using SAMtools (version 0.1.19, option: depth) [47].

The scaffolds were ordered using mauve (snapshot 2013-06-07, default parameters) (Darling et al., 2010) based on the most closely related reference genome from the dendrogram of **Figure 2B**.

*Ab initio* annotation was performed using Prodigal (version 2.60, default parameters) (Hyatt et al., 2010), which is part of the rapid prokaryotic genome annotation software (Prokka, default parameters) (Seemann, 2014).

### Gene-Trait Matching

All CDSs of the different strains were clustered into OGCs using OrthoMCL (version 2.0.9, default parameters) (Li et al., 2003). Every OGC was tested for significant association with the growth phenotype in medium devoid of cysteine. Therefore, the presence or absence of every OGC was counted for the strains that grew and did not grow in medium devoid of cysteine, respectively. The *p*-values were calculated using Fisher’s exact test (Fisher, 1922). The resulting *p*-values were corrected for multiple testing using the Benjamini–Hochberg method (Benjamini and Hochberg, 1995).

### Search of the *cysK-ctl-cysE* Gene Cluster in Other Species

To find the *cysK-ctl-cysE* gene cluster, the translated CDS sequences of the three genes of strains FAM18149 were aligned against the non-redundant nucleotide sequence of NCBI (March 2016) using tblastn (version 2.6.0+, default parameter) (Altschul et al., 1990). To find the *cysK-ctl-cysE* gene cluster in other genomes two selection steps were performed. First, the genome must have homology to all three genes within a window of 20 kbp. Second, the homologous parts must have the same gene order as the cluster in *L. paracasei*. We aligned all sequence using MAFFT (version 7.187, default parameters) (Sievers et al., 2011). Finally, we computed the phylogenetic tree using RaxML (version 8.2.9, options: -f a -m GTRGAMMA -# 1000) (Stamatakis, 2006) based on the merged multiple alignments.

### Phylogenetic Trees and Orthologs Detection

Orthologous gene clusters were build using Roary (version 3.6.2, default parameters) (Page et al., 2015).

We selected the corresponding CDSs from the core genome GCs that contained only a single ortholog from each of the studied bacterial strains. We aligned these CDSs of all GCs using MAFFT (version 7.187, default parameters) (Sievers et al., 2011). Finally, we computed the phylogenetic tree using RaxML (version 8.2.9, options: -f a -m GTRGAMMA -# 1000) (Stamatakis, 2006) based on the merged multiple alignments.

### Graphical Representation

The phylogenetic trees were created using iTOL (Letunic and Bork, 2007). Plot and graphs were created with ggplot2 (Wickham, 2010).

## Ethics Statement

Ethical approval was not required for our study as we exclusively used natural bacterial strains.

## Author Contributions

All authors conceived and designed the study, and read and approved the final manuscript. SI and HB performed experiments. DW and RB performed bioinformatics analyses. DW, SI, and RB wrote the manuscript.

## Conflict of Interest Statement

The authors declare that the research was conducted in the absence of any commercial or financial relationships that could be construed as a potential conflict of interest.

## Footnotes

1 https://www.ncbi.nlm.nih.gov/bioproject/?term=PRJNA295910

2 http://www.ncbi.nlm.nih.gov/genbank/

## Abbreviations

BLAST: Basic Local Alignment Search Tool
CDS: coding sequence
LAB: lactic acid bacteria
OGC: orthologous gene cluster build by OrthoMCL
GC: Orthologous Gene Cluster build by Roary
ORF: open reading frame.
